# Exo70 Subunit of the Exocyst Complex Is Involved in Adhesion-Dependent Trafficking of Caveolin-1

**DOI:** 10.1371/journal.pone.0052627

**Published:** 2012-12-27

**Authors:** Maud Hertzog, Pedro Monteiro, Gaëlle Le Dez, Philippe Chavrier

**Affiliations:** 1 Membrane and Cytoskeleton Dynamics, Institut Curie, Research Center, CNRS- UMR144, Paris, France; University of Birmingham, United Kingdom

## Abstract

Caveolae are specialized domains of the plasma membrane, which play key roles in signaling, endocytosis and mechanosensing. Using total internal reflection fluorescent microscopy (TIRF-M), we observe that the exocyst subunit Exo70 forms punctuate structures at the plasma membrane and partially localizes with caveolin-1, the main component of caveolae. Upon cell detachment, we found that Exo70 accumulates with caveolin-1-positive vesicular structures. Upon cell re-adhesion, caveolin-1 traffics back to the plasma membrane in a multistep process involving microtubules and actin cytoskeleton. In addition, silencing of Exo70 redirects caveolin-1 to focal adhesions identified by markers such as α5 integrin or vinculin. Based on these findings, we conclude that Exo70 is involved in caveolin-1 recycling to the plasma membrane during re-adhesion of the cells to the substratum.

## Introduction

Caveolae are bulb-shaped pits present in several mammalian cell types including adipocytes and muscle cells [1], [2]. These structures play key roles in compartmentalization and organization of signaling pathways for cell growth and differentiation. In addition, caveolae were recently implicated in membrane-mediated mechanical responses [3], [4]. Caveolin-1 (Cav1) is the main component of caveolae. Cav1 adopts a hairpin-like shape within the membrane bilayer with both the N and C-terminus facing the cytoplasm [1]. Recent studies showed that the Cav1 partner protein, Polymerase I and Transcript Release Factor (PTRF)/cavin-1 selectively associates with mature caveolae at the plasma membrane and is involved in caveolae formation and function [1], [5]. Total internal reflection fluorescence microscopy (TIRF-M) allowed characterizing the dynamics of individual caveolae and revealed that caveolae can be stored in stationary multi-caveolar structures at the plasma membrane, or undergo kiss and run processes without disassembling the caveolar coat [6]. Moreover, caveolae can undergo long-range cytoplasmic transport during diverse regulated processes such as mitosis and during loss of integrin-based adhesion to the extracellular matrix (ECM) [1], [7], [8]. All together, these data suggest some interplay between caveolar trafficking and cell adhesion [9].

Initially identified in *Saccharomyces cerevisiae*, the exocyst complex comprises eight subunits named Sec3, Sec5, Sec6, Sec8, Sec10, Sec15, Exo70 and Exo84 [10], [11]. This complex controls the docking and insertion of secretory and endocytic recycling vesicles to growing regions of the plasma membrane and impinges on diverse cellular processes requiring polarization of membrane trafficking such as cell motility and neuronal development [12], [13]. In yeast, Exo70 interacts with phospholipids at the plasma membrane and is crucial for tethering of secretory vesicles for exocytosis [14]. Phospholipid binding and plasma membrane localization are conserved in mammalian cells suggesting a similar role of Exo70 in targeting of the exocyst complex to the plasma membrane and regulation of vesicle docking [14]–[16]. Previous studies showed that detachment of cells from integrin-mediated adhesion triggers the caveolin-dependent internalization of lipid rafts [9]. Conversely, re-adhesion of cells to the ECM induces the re-insertion of lipid rafts to the plasma membrane in a process that involves the Ras-related GTP binding proteins RalA and ARF6 and their downstream effectors, exocyst complex [17], [18], [19]. However, the specific contribution of Exo70 to Cav1 trafficking remains largely unknown.

Here, using TIRF-M we could show that Exo70 accumulates in punctuate plasma membrane domains where it co-localized with Cav1. Upon cell detachment, Exo70 localized to Cav1-positive intracellular compartments. When cells were allowed to re-adhere, silencing of Exo70 led to the accumulation of Cav1-positive structures at the cell periphery close to focal adhesions (FAs), suggesting that loss of Exo70 function results in Cav1 mistargeting during cell spreading.

## Results

### Exo70 co-localizes with Cav1 at the plasma membrane

**Figure 1:**
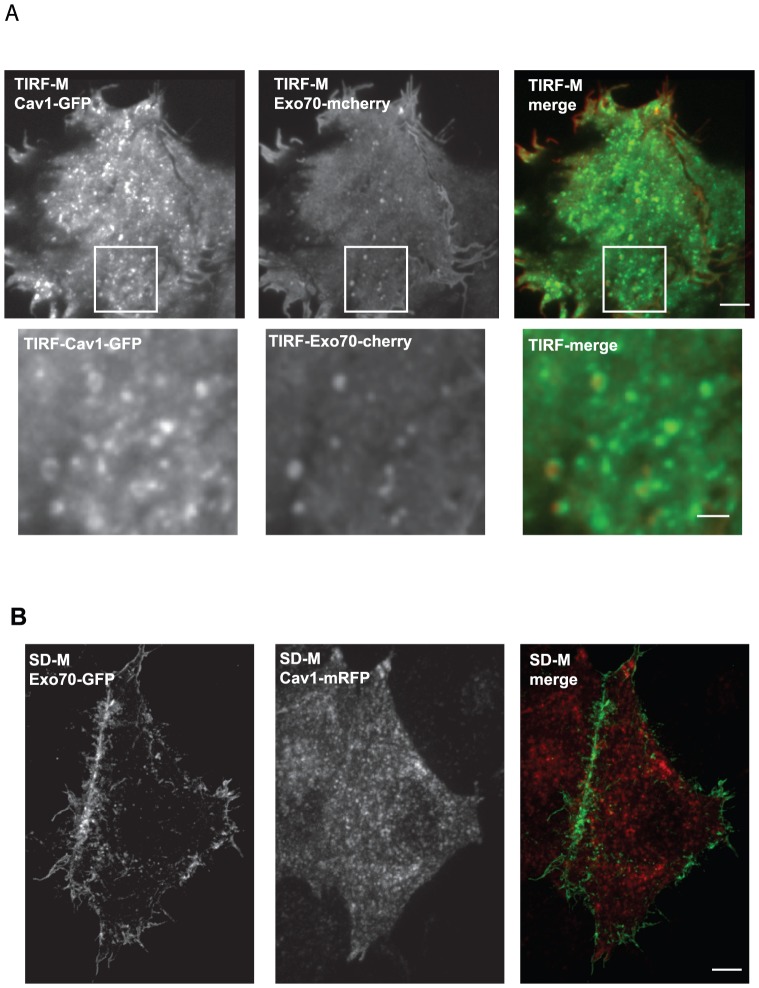
Partial co-localization and co-trafficking of Exo70 and Cav1 at the plasma membrane. (**A**) Hela cells expressing Cav1-GFP and Exo70-mCherry were visualized by TIRFM (upper panel); Scale bar, 5 µm. Higher magnification of the boxed region is shown (bottom panel). Scale bar, 1 µm. (**B**) Hela cells expressing Cav1-mRFP and Exo70-GFP were visualized using time-lapse confocal spinning disk microscopy. Scale bar, 5 µm.

TIRF-M revealed a punctuate distribution of Exo70-mCherry on the adherent surface of Hela cells and co-localization with a subset of Cav1-GFP-positive structures (representing 20 to 30% of total Cav1-positive structures) and probably corresponding to caveolae (Fig. 1A and S1A). Live-cell confocal spinning disk microscopy confirmed this punctuate pattern and plasma membrane localization of Exo70-GFP whereas Cav1-mRFP associated predominantly with intracellular vesicular structures (Fig. 1B). It has been previously reported that Exo70 binds directly to phospholipids and that binding to PtdIns(4,5)P2 mediates interaction of Exo70 with the plasma membrane [14], [16]. When we used the PtdIns(4,5)P2-binding protein tubby [20], tubby-GFP was present in plasma membrane punctuate structures observed by TIRF-M, which partially localized with Exo70-mCherry (Fig. S1B) indicating that Exo70 localization sites at the plasma membrane are enriched in PtdIns(4,5)P2.

**Figure 2:**
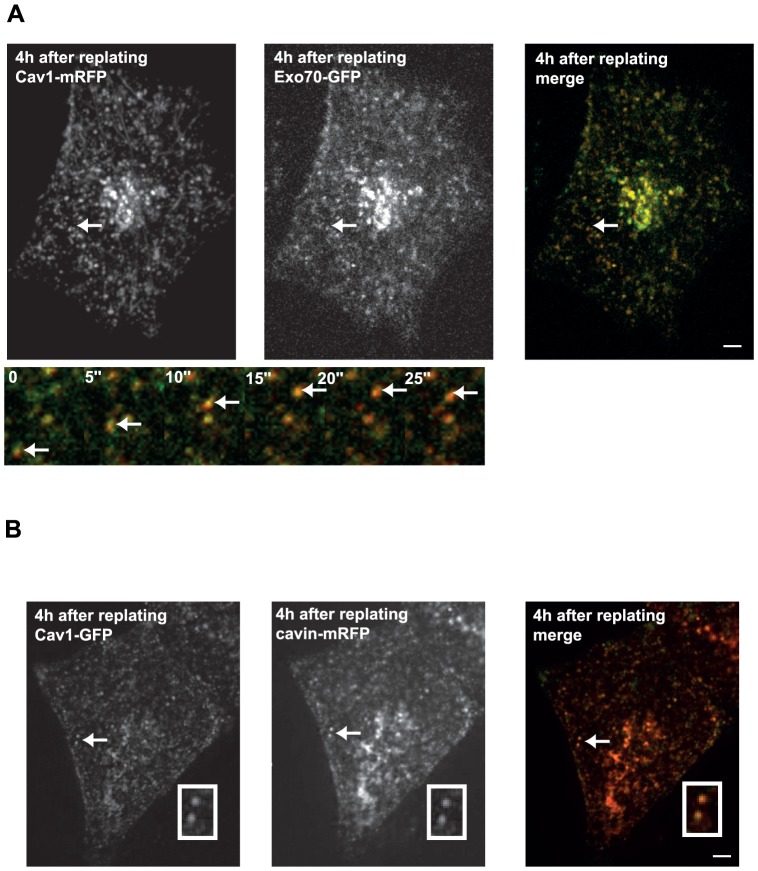
Exo70 redistributes in Cav1-positive compartments upon cell detachment. (**A**) Hela cells expressing Cav1-mRFP and Exo70-GFP were kept in suspension for 1 h and replated on fibronectin for 3 h, and then visualized by confocal dual-colour spinning-disk microscopy (see corresponding movie S1). Arrow points to a dynamic Cav1- and Exo70-positive vesicle. Bottom panel shows selected frames from the time-lapse series corresponding to the boxed region in the upper panel. Time is given in second. (**B**) Hela cells expressing Cav1-GFP and cavin-1-mRFP were treated as in panel A. Inset shows higher magnification of region indicated by an arrow. Scale bars, 5 µm.

Caveolae internalization can be triggered by several stimuli, including loss of integrin- mediated cell adhesion [21]. Upon cell detachment, Cav1-positive PM domains initiate an inward traffic to the perinuclear area. Hela cells expressing Cav1-mRFP and Exo70-GFP were detached from the substratum by trypsinization, maintained for 1 hr in suspension, replated onto fibronectin (FN)-coated substrate and visualized by spinning disk microscopy. Cav1-mRFP was observed in dynamic vesicles that shuttled between the perinuclear region and the cell periphery (Fig. 2A and movie S1). Analysis of the distribution and dynamics of Exo70-GFP during cell replating revealed that Exo70 was strongly associated with Cav1-positive vesicles throughout the cell (compare Fig. 1B and 2A). Thus, these results reveal a dynamic association of Exo70 with Cav1 transport intermediates following cell detachment and re-adhesion. In addition to Cav1, cavin-family proteins are required for caveolae formation and PTRF/cavin-1 associates with mature caveolae [22]. Interestingly, cavin-1 partially redistributed with Cav1 in intracellular vesicles in cells replated on FN-coated substrates after detachment, suggesting that mature caveolae are internalized and trafficked upon cell detachment (Fig. 2B). In addition, we acquired time-lapse sequences of HeLa cells expressing either Cav1-GFP and Exo70-mCherry or Cav1-mRFP and Exo70-GFP by TIRF-M (data not shown). These movies were acquired starting 1, 2, or 3 hours after cell detachment and replating. In all cases, we observed that 100% of Exo70-positive structures were Cav1-positive, while only a subset (20–30%) of Cav1-positive structures co-localized with Exo70 (data not shown).

### Actin and microtubule cytoskeletons are involved in distinct steps of Cav1 trafficking

**Figure 3:**
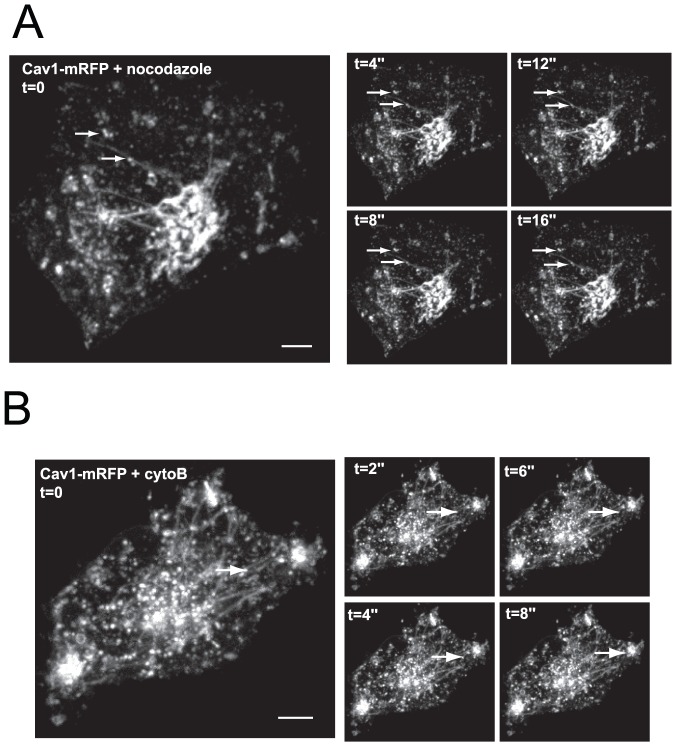
Actin and microtubule cytoskeletons act at distinct steps of Cav1 trafficking. (**A, B**) Hela cells expressing Cav1-mRFP maintained in suspension for 1 h, were replated on fibronectin for 3 h in the presence of 10 µM nocodazole (panel A) or 10 µg/ml cytochalasin-B (panel B) for 30 min. Cells were then analyzed by time-lapse confocal spinning disk microscopy. The right panels represent selected frames from the time-lapse series (time is given in second). Arrows point to Cav1-positive intracellular vesicles. See corresponding movie S2 (panel A) and movie 3 (panel B). Scale bars, 5 µm.

To identify mechanisms underlying cell detachment-induced Cav1 redistribution, we investigated the involvement of cytoskeleton components using nocodazole and cytochalasin-B (cytoB), which destabilize microtubule and actin networks, respectively. Treatment with nocodazole induced a blockade of Cav1 transport, which accumulated in a network of static tubulo-vesicular extensions extending towards the cell periphery (Fig. 3A, movie S2), indicating that recycling of Cav1 to the surface is a MT-dependent process. Inhibition of actin assembly by treatment with cytoB induced the redistribution of Cav1 to small, scattered vesicles in the central region of the cells and in some peripheral accumulations at the cell edges (Fig. 3B, movie S3). Remarkably, a network of radially extending tubular membranes connected the perinuclear vesicles and the peripheral Cav1 pools. Collectively, and in agreement with previous reports [23], [24], these observations suggest that both MTs and actin cytoskeleton are involved in distinct steps of Cav1 trafficking: (i) a MT-dependent long range transport from perinuclear endosomal compartments that we identified as late endosomes (LEs) by co-localization with GFP-rab7 and GFP-VAMP7 (see Figure S2) (ii) and an actin-dependent step involved in Cav1 trafficking at the cell periphery.

### Silencing of Exo70 leads to an accumulation of Cav1 in focal adhesions

**Figure 4:**
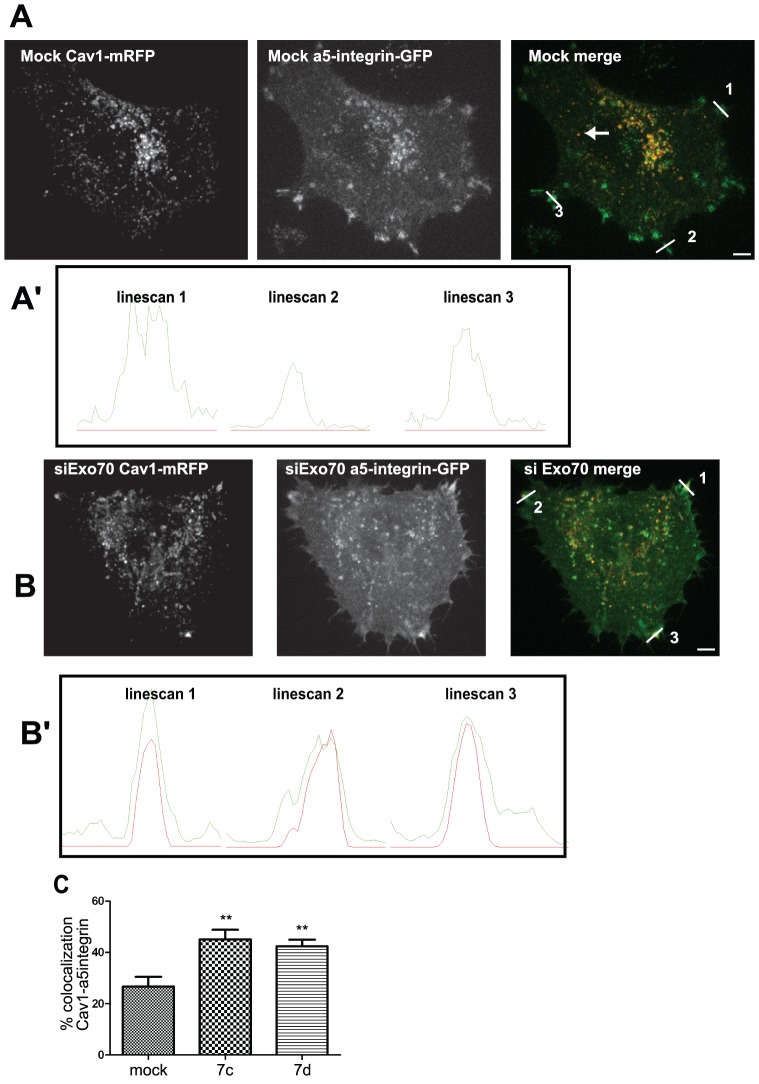
Exo70 is required for Cav1 transport to the plasma membrane. (**A, B**) Hela cells expressing Cav1-mRFP and α5-integrin-GFP either mock-treated (A) or treated with a specific siRNA to silencing Exo70 (B) were maintained in suspension for 1 h and replated on fibronectin for 3 h, and visualized using time-lapse spinning disk microscopy. Scale bars, 5 µm. (**A′, B′**) Intensity profile of Cav1 (red) and α5-integrin (green) along the white lines shown in panel A and B. (**C**) Co-localization analysis of Cav1-mRFP and α5-integrin-GFP in cells as in panels A and B in a 20-pixel width region along the cell periphery of mock- or Exo70 siRNA-depleted cells using two specific siRNAs (siRNA 7c and 7d). Results are the average of mean percentage of co-localization ± s.e.m. of three experiments. ** *P*<0.05. Experiment has been repeated three times; with number of cell analysed: n = 19 for mock cells, n = 17 for siRNA 7c, and n = 17 for siRNA 7d.

Cav1 is internalized together with integrins upon cell detachment and β1 integrin regulates Cav1 trafficking and recycling to the plasma membrane for caveolae reassembly upon cell adhesion [25], [26]. Consistent with these findings, we observed a partial co-localization of α5-integrin-GFP with Cav1-mRFP in cytoplasmic vesicles upon cell detachment and re-adhesion (Fig. 4A). In addition, α5-integrin-GFP associated with reforming focal adhesions at the cell edge, which were negative for Cav1 (Fig. 4A and A′). Noticeably, in 20% of the cells, Cav1-mRFP and α5-integrin-GFP associated with membrane tubules extending radially from the perinuclear compartments, and frequently targeting newly reformed focal adhesions (movies S3 and S4). All together, these findings indicate that Cav1 and integrins traffic together in tubulo-vesicular transport carriers to the cell periphery.

**Figure 5:**
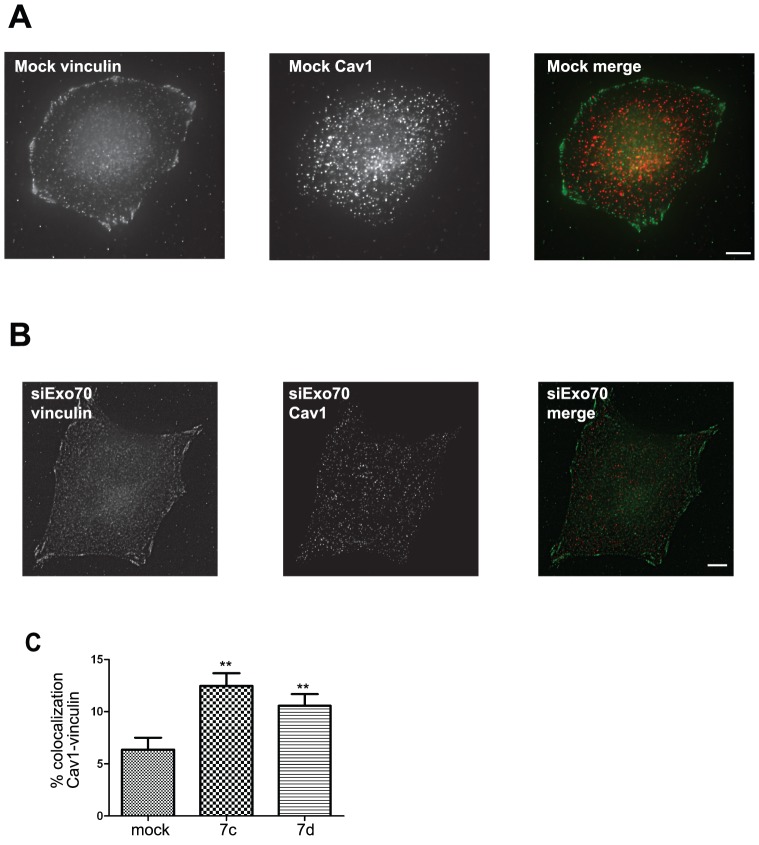
Silencing of Exo70 leads to Cav1 accumulation in focal adhesions. Mock-treated (**A**) or Exo70-depleted cells (**B**) were detached for 1 h and replated on fibronectin-coated substrate for 3 h, and then fixed and stained for endogenous vinculin and Cav1. Scale bars, 5 µm. Co-localization between Cav1 and vinculin was quantified in a 20-pixel width region from the cell periphery and compared in Exo70-depleted with two independent siRNAs *vs*. mock-treated cells (**C**). Results in panel C are the average of mean percentage of co-localization ± s.e.m. of three experiments. ** *P*<0.05. Experiment has been repeated three times with number of cells analysed: n = 25 mock cells, n = 9 for cells treated with Exo70 siRNA 7c, and n = 23 for siRNA 7d.

We investigated whether Exo70 may play a role in Cav1 trafficking and recycling upon cell adhesion [27]. Silencing of Exo70 using two independent siRNAs (Fig. S3) inhibited cell spreading on fibronectin-coated substratum during the early phase of replating (3 to 6 hrs after replating) (Fig. S4), Silencing of Exo70 caused the accumulation of Cav1-mRFP to peripheral α5-integrin-positive structures with a morphology typical of focal adhesions (Fig. 4, compare panels A and B and linescan profiles in panel A′ and B′). Co-localization of Cav1 and α5-integrin was quantified and compared between mock- and siExo70-treated cells in a peripheral region of the cells enriched in focal adhesions (see Material and Methods section) (Fig. 4C). In mock-treated cells, we observed a 26.6% co-localization of Cav1-mRFP and α5-integrin-GFP in the peripheral region. This percentage increased to 42–45% in cells depleted for Exo70 with two-independent siRNAs (Fig. 4C). These results suggest that, in the absence of Exo70, Cav1 (or Cav1-positive vesicles) is retained in focal adhesions. Next, we addressed the effect of Exo70 knockdown on the distribution of endogenous Cav1 protein. Hela cells were detached and maintained in suspension for 60 min before replating on fibronectin for 3h, and labeled for endogenous Cav1 and the focal adhesion protein vinculin. Using the quantification method described above, we found a 6.3% co-localization of Cav1 and vinculin at focal adhesions. This value increased to 10–12% in cells depleted for Exo70 (Fig. 5). Thus, these results suggest that depletion of Exo70 results in an accumulation of Cav1 at focal adhesions.

## Discussion

This study reveals that Exo70 accumulates in dotty structures, which are visible by TIRF-M at the plasma membrane. Exo70-positive plasma membrane domains are enriched in PtdIns(4,5)P2, a lipid known to mediate binding of Exo70 at the plasma membrane both in yeast and mammal cells [14], [16]. In addition, in mammalian cells Exo70 has been shown to recruit other exocyst components to the plasma membrane and to control the tethering of secretory vesicles [15]. Thus, Exo70-positive puncta that we observed at the adherent plasma membrane of HeLa cells may represent some exocytic vesicles docking sites. Interestingly, these puncta also appear to be enriched for the main caveolar component, Cav1.

Whether caveolae can mediate endocytosis of specific cargo(es) is still a matter of debate [1]. In contrast, several studies have documented the internalization of caveolae in internal compartments during diverse cellular processes including mitosis and cell detachment [7], [28]. Cell adhesion signaling through ß1 integrins seems to play a key role for the recycling of caveolar components and the formation of caveolae at the plasma membrane [6], [20], [23], [24]. Keratinocytes from mice knockout for ß1 integrin or integrin-linked kinase (ILK) show a very low level of caveolae due to impaired traffic of Cav1-containing transport vesicles along microtubules [23], [24]. ILK was shown to promote the recruitment of actin regulators allowing stable insertion of caveolae into the plasma membrane [24]. Our study confirms that intracellular transport of Cav1 is a multistep process controlled by cell adhesion. First, cell detachment leads to a massive redistribution of Cav1 and α5ß1 integrins in a perinuclear compartment. This compartment is rab7- and Vamp7-positive suggesting that upon cell detachment, Cav1 is internalized and trafficked to late endosome/lysosomal compartments. This is reminiscent of the effect of cholesterol depletion or serum deprivation, which trigger caveolae accumulation into late endosomes/lysosomes [29]. Members of the cavin protein family, which comprise four proteins including cavin-1/PTRF are also crucial for caveolae formation [5], [22]. Here, we observed that Cav1 partially redistributed with cavin-1 after cell detachment, suggesting that mature caveolae are internalized and traffic upon cell detachment (figure 2B). A recent study supports the conclusion that under normal conditions, upon docking and fusion of Cav1-positive transport carriers with the plasma membrane at nascent focal adhesions, Cav1 would be segregated away from focal adhesion to redistribute laterally along the plasma membrane bilayer for caveolae assembly [30]. Our data indicate that in adherent cells, at steady-state, Exo70 domains co-localize with a subset of Cav1-positive structures at the plasma membrane, very likely corresponding to caveolae, while these proteins show no association in the cytoplasm. Upon cell detachment and readhesion, Cav1 redistributes to late endosomes and strongly co-localizes with Exo70. Upon silencing of Exo70, Cav1 (or Cav1-positive transport carriers) are retained at focal adhesions and mistargeted, suggesting that Exo70 is involved in Cav1 recycling to the plasma membrane during readhesion of the cells to the substratum.

To conclude, functions for caveolae have been identified in biomechanical remodeling or mechanosensing [3], [4], [28]. Indeed, Cav1 and caveolae are abundant in cells experiencing high mechanical stress such as muscle, skin and endothelial cells. The fact that Cav1 and caveolae levels at the cell surface are regulated by cell adhesion provide a rational for the regulation of caveolae assembly by mechanical clues.

## Materials and Methods

### Cell Culture, transfection and siRNA treatment

Hela cells were cultured in DMEM medium (GIBCO) containing 15% FCS and 2 mM glutamine at 37°C and 5% CO_2_. For treatment with cytoskeleton-disassembling drugs, cells were treated for 30 min with nocodazole (working concentration 10 µM) or cytochalasin-B (10 µg/ml). Drugs were purchased from Sigma.

Cells were transfected with following plasmids: Exo70-GFP or -mCherry, Exo84-GFP or -mCherry cloned in pcDNA3.1 plasmid; Cav1-GFP or mRFP constructs were a kind gift of Dr A. Helenius (ETH, Zürich) [31]; Cavin-1-mRFP was a kind gift of Dr C. Lamaze (Institut Curie, Paris) [3] and tubby-GFP was a kind gift L. Shapiro (Columbia University, New York) [20]. Transfections with plasmid DNA (1 µg) were carried out with Fugene (Roche) according to the manufacturer' instructions. Cells were analyzed 24 h after transfection.

Cells were transfected with two independent siRNAs (Dharmacon) specific for EXOC7 (Exo70) at a concentration 100 nM using Oligofectamine (Invitrogen). Sequences of the forward strand were as follows: EXOC7-duplexC: 5′-GAGAUGACAUGCUGGACGUUU-3′, EXOC7-duplexD: 5′-GGAAGGCCAUUGUGCGACAUU-3′. Mock or siRNA-treated cells were analyzed 3 days after transfection.

### Western blot and antibodies

siRNA-treated cells were lysed in lysis buffer (50 mMTris-HCl [pH 7.4], 150 mM NaCl, 1% Triton X-100, 1 mM EDTA, and protease inhibitors Mini complete, Roche) and centrifuged at 16,000 g for 10 min at 4°C. Supernatants were separated by SDS-PAGE and analyzed by immunoblotting. Rabbit polyclonal antibodies against actin (A5060) and Cav1 (610060) were purchased from Sigma and BD Bioscience, respectively. Monoclonal antibody against EXOC7 was a generous gift of Dr S. Hsu (Rutgers University, NJ). Monoclonal against human vinculin was a kind gift of Dr Marina Glukhova (Institut Curie-Paris). HRP-conjugated anti-mouse IgG, Cy3-conjugated anti-mouse and Cy5-conjugated F(ab)2 anti-mouse antibodies were purchased from Jackson ImmunoResearch Laboratories. Alexa-Fluor-labeled anti-mouse IgG antibodies were purchased from Molecular Probes (Invitrogen).

### Caveolin1 trafficking assay and quantification

Hela cells transfected with the indicated constructs were detached, held in suspension in culture medium supplemented with 20 mM Hepes at 37°C for 1 h and replated on fibronectin (10 µg/ml) coated glass dish. After the indicated time of replating, the cells were fixed for immunfluorescence analysis or observed in live using spinning disk or TIRF-M.

For quantification of co-localization between α5-integrin-GFP and Cav1-mRFP at the cell periphery, the area of cell surface was drawn with the Threshold command of MetaMorph 7. A 20-pixel width region from the cell periphery was created using both the Erode and Create Region commands of MetaMorph 7. The percentage of co-localization of the two proteins was measured using the Measure Colocalization command of MetaMorph 7 in the 20-pixel region. Statistical analyses were performed using Student's *t* test in GraphPad Prism 5 software.

### Live cell imaging by TIRF and spinning disk confocal microscopy

For live cell imaging by TIRF-M, HeLa cells seeded onto glass-bottom dish were transfected with the indicated constructs and imaged the next day with a 100× 1.49 NA TIRF objective on a Nikon TE2000 (Nikon France SAS, Champigny sur Marne, France) inverted microscope equipped with a QuantEM EMCCD camera (Roper Scientific SAS, Evry, France/Photometrics, AZ, USA), a dual output laser launch which included 491 and 561 nm 50 mW DPSS lasers (Roper Scientific), and driven by Metamorph 7 software (MDS Analytical Technologies). A DV2 beam-splitter system (Roper Scientific/Photometrics) mounted on the light path enabled the simultaneous acquisition of the two emission channels. A motorized device driven by Metamorph allowed accurate positioning of the illumination light for evanescent wave excitation.

For spinning disk microscopy, HeLa cells plated onto a glass-bottom dish coated with fibronectin (Sigma, 10 µg/ml) and transfected with the indicated constructs. Images were acquired with 100 ms exposure time at 2 or 5 s interval as indicated using a spinning disk microscope based on a CSU22 Yokogawa head mounted on the lateral port of an inverted microscope Leica IRE2 equipped with a 100× 1.4NA Plan-Apo objective and a dual output laser launch which included 491 and 561 nm ERROL laser bench 491 nm, 561 nm (Roper Scientific). Images were acquired with a Camera EMCCD Cascade 512×512 (Photometrics). The system was steered by Metamorph 7 software.

### Immunofluorescence analysis

For immunofluorescence analysis, Hela cells were plated on fibronectin coated coverslips, fixed and extracted with 0.3% Triton X-100 in 4% PFA for 20 min and further fixed for 20 min by 4% PFA. Then, cells were incubated with Cav1 antibodies in PBS and washed with PBS. Bound antibodies were detected with Cy3-conjugated mouse antibodies. Cells were then mounted in ProLong Gold antifade reagent (Invitrogen) containing DAPI. Images were taken using Eclipse 90i Upright Microscope with a CCD Camera CoolSNAP HQ2 and a Piezo Flexure Objective Scanner. The system was steered by Metamorph 7 software.

## Supporting Information

Figure S1 **TIRF-M revealed specific structures positive for Cav1-GFP and Exo70-mCherry.** (**A**) Hela cells expressing Cav1-GFP and Exo70-mCherry were visualized by TIRF-M (top panel). The bottom panel shows a wide-field image of the same field. **B**) Hela cells expressing tubby-GFP and Exo70-mCherry were visualized by TIRF-M. Scale bars, 5 µm. Inset shows higher magnification of region indicated by an arrow.
(EPS)

Figure S2 **Caveolin1 co-localizes with late endosomes markers, GFP-rab7 or GFP-VAMP7.** (**A**) Hela cells expressing Cav1-mRFP and GFP-rab7 were detached and maintained in suspension for 1 h in culture medium at 37°C, and then replated on fibronectin for 3 h and visualized using time-lapse confocal spinning disk microscopy (upper panel). The lower panels represent selected frames from the time-lapse series (time is given in second). Arrows point to a Cav1-, rab7-positive intracellular vesicle. (**B**) Hela cells expressing Cav1-mRFP and GFP-VAMP7 were treated and analyzed as in panel A. Scale bars, 5 µm.
(EPS)

Figure S3 **Exo70 silencing.** Samples of Hela cells treated with two independent Exo70 siRNAs (7c and 7d) were analyzed by western blotting with the indicated antibodies. Levels of Exo70 were reduced by 1.9 and 2.2-fold upon treatment with Exo70 siRNA 7c and 7d, respectively. Actin immunoblotting staining was used as a loading control. Levels of Exo70 strongly decreased without affecting Cav1 levels.
(EPS)

Figure S4 **Silencing of Exo70 inhibited cell spreading on fibronectin-coated substratum.** Mock-treated cells or cells silenced for Exo70 were maintained in suspension for 60 min and replated on fibronectin for 3 or 6 h and fixed. The projected cell surface area was measured using Metamorph software. Graph represents the mean projected cell surface area ± S.E.M. in µm^2^ measured before putting cell in suspension (t = 0); after 3 h (t = 3 h) and 6 h of replating (t = 6 h) on fibronectin coated substrates. ** *P*<0.05.
(EPS)

Movie S1 **Microtubule disassembly interferes with Cav1-positive vesicle trafficking.** Hela cells expressing Cav1-mRFP were kept in suspension for 1 h, and then replated on fibronectin-coated substrate for 3 h and further incubated in the presence of nocodazole for 30 min. Cells were visualized using time-lapse spinning disk microscopy. Images were taken every 2 s. Under Nocodazole treatment, Cav1- positive vesicles are static and concentrated in the cell center.
(MOV)

Movie S2 **Cytochalasin-B treatment interferes with Cav1 trafficking.** Hela cells expressing Cav1-mRFP are put in suspension for 1 h replated on fibronectin-coated substrates for 3 h, incubated with 10 µg/ml cytochalasin-B for 30 min, and visualized using time-lapse Spinning Disk Microscopy. Images are taken each 2 s. Under these conditions, an accumulation of Cav1-mRFP positive vesicles appeared at the cell periphery.
(MOV)

Movie S3 **Cav1-positive tubules target peripheral focal adhesions.** Hela cells expressing Cav1-mRFP and α5-integrin-GFP are put in suspension for 1 h replated on fibronectin-coated substrates for 3 h, and visualized using time-lapse confocal spinning disk microscopy. Images are taken each 5 s.
(MOV)

Movie S4 **Cav1-positive tubules target peripheral focal adhesions.** Hela cells expressing Cav1-mRFP and α5-integrin-GFP are put in suspension for 1 h replated on fibronectin-coated substrates for 3 h, and visualized using time-lapse confocal spinning disk microscopy. Images are taken each 5 s.
(MOV)
