# Correction: Exo70 Subunit of the Exocyst Complex Is Involved in Adhesion-Dependent Trafficking of Caveolin-1

**DOI:** 10.1371/annotation/fc7791c5-45e2-44ae-868b-f0529c56033f

**Published:** 2013-08-23

**Authors:** Maud Hertzog, Pedro Monteiro, Gaëlle Le Dez, Philippe Chavrier

Additional institutional affiliations for the first and third authors were incorrectly omitted. Maud Hertzog is also affiliated with Laboratoire de Microbiologie et Génétique Moléculaire, LMGM-CNRS UMR 5100, Université Paul Sabatier, Toulouse, France, and Gaëlle Le Dez is also affiliated with CNRS, UMR 6061, Institut Génétique et Développement de Rennes, Université Rennes 1, UEB, IFR 140, Rennes, France. 

